# Severe burn injury in late pregnancy: a case report and literature review

**DOI:** 10.1186/s41038-015-0002-z

**Published:** 2015-05-28

**Authors:** Yan Shi, Xiong Zhang, Bo-Gao Huang, Wen-Kui Wang, Yan Liu

**Affiliations:** Department of Burn and Plastic Surgery, Shanghai Jiaotong University, School of Medicine Affiliated Ruijin Hospital, Shanghai, 200025 China

**Keywords:** Burn, Pregnancy, Fluid resuscitation

## Abstract

The management of serious burn injuries during pregnancy is an unsolved clinical problem because of the low incidence of this disease. Although it has been documented that the effect of burns on fetal and maternal survival is detrimental, there have been conflicting reports among the different burn centers regarding the mortality of burned pregnant women and the management of burn patients during pregnancy. We report a case of severe burn in late pregnancy treated at our burn center. Additionally, we searched and summarized the literature concerning the management of pregnant patients to provide useful information for their treatment.

## Introduction

The management of serious burn injuries during pregnancy needs to be better studied and standardized due to the low incidence of this disastrous condition. In general, severe burn injury in pregnant patients is more common in developing countries because most of the case reports have come from low-income countries [1-4]. The maternal mortality and morbidity in the literature concerning severe burn injury in pregnant patients are usually significantly higher than those in the general population with the same degree of injury, although there are tremendous differences among different reports [3,5]. As early as the 1990s, Rode et al., reported that when over 50% of the total body area was burned, the mother’s survival was unlikely [6]. Since then, the mortality and morbidity rates have not changed. In a 9-year prospective study conducted on 51 pregnant women with a severe burn injury, the authors reported that both the maternal and fetal mortality rates reached 100% in the pregnant women with the total burned body surface area exceeding 40% total body surface area (TBSA) [7]. Recently, however, in a 6-year cross-sectional study, 39 (1.88%) women were found to be pregnant among 2,067 women with a severe burn injury, and the mortality rate of all the pregnant women reached 66.7% with more than 50% death rate when the burn area exceeded 60% [8]. It is widely recognized that the effect of burns on fetal and maternal survival is detrimental, and there is a positive relationship between the percentage of maternal total body burn area and maternal and fetal mortality, as well as premature delivery [9]. However, Amy et al. [10] reported that pregnancy does not alter the maternal outcome after thermal injury. Because there were conflicting reports among different burn centers and an enormously high mortality in pregnant women with severe burn injury, collecting more information and reaching a consensus on the management of burns in pregnancy are necessary.

## Case report

The patient was a 28-year-old woman who was referred to our Burn Institute 1.5 h after a flame burned her head, trunk, and limbs. The patient had a distressed facial expression and was cooperative in the physical examination. She had no breathing difficulty, although obvious swelling, particularly on the head, was observed. No ashes or blisters were found on the oropharyngeal mucosa by laryngoscopy examination. Physical examination data on admission were as follows: body weight, 56 kg; blood pressure, 132/67 mmHg; pulse rate, 88 bpm; body temperature, 36.8°C; and respiratory rate, 15 bpm. The burn wounds were distributed on the face, neck, trunk, and limbs, and the total burn area was 50% TBSA. Wounds were presented as red, or red and white in appearance, and parts of them were dry with diminished sensation. The laboratory results were as follows: blood routine examination: WBC, 18.64 × 10^9^/L; neutrophil granulocyte, 90.3%; Hgb, 105 g/L; RBC, 4.03 × 10^12^/L; Hct, 0.329; platelet, 296 × 10^9^/L. The liver and kidney function indexes were all in the normal range except that the total protein level was 50 g/L and the albumin level was 24 g/L. The obstetric examination results were as follows: The patient was 35 weeks and 5 days pregnant, the uterine fundus was halfway between the umbilicus and xiphoid process, and the fetal outline was palpable. No vaginal bleeding or discharge was observed, and uterine contraction was not palpable. Fetal movements were occasionally felt. The cervix was not open, and the fetal membranes had not broken. The fetal heart rate (FHR) was 150 bpm. There was no gestational hypertension, gestational diabetes, or other combined diseases reported. The admission diagnosis was that the flame burned 50% TBSA (superficial second-degree 20% TBSA, deep partial thickness burn 30% TBSA) with a third-trimester pregnancy.

During emergency treatment, lactate Ringer’s solution was infused for fluid resuscitation, and 1,500 mL of lactate Ringer’s solution was infused during the first 9 h after injury. The burns were covered with Vaseline gauze after simple wound cleaning. Nine hours after the injury, after consulting and discussing with the obstetrician, a cesarean section was performed under spinal epidural anesthesia, and a live baby girl was delivered. The surgery lasted approximately 1 h, and the blood pressure was stable during the surgery. The Hgb level after the surgery was 97 g/L. The baby had a body weight of 2,220 g and a body length of 46 cm with APGAR scores of 10’, 10’, and 10’. The vital signs of the patient were stable during surgery. After delivery of the baby, intravenous fluid resuscitation was continued for the patient using the Ruijin (Resuscitation) formula. Ruijin’s formula involves the infusion of a crystalloid solution, which comprised primarily lactated Ringer’s solution and/or colloids, usually plasma, at a rate of 1.5 mL/kg/percentage of the burned body surface area for the first 24 h and half of the amount of the actually infused crystalloid and colloid solution for the second 24 h. In addition, 2,000~3,000 mL of physical-demanding water was applied every 24 h after injury. The type and volume of the solution delivered for liquid resuscitation are summarized in Table 1. The hourly urine output was 50~350 mL during the first 48 h after the burn injury. The treatment for the patient included the application of silver sulfadiazine cream on the wounds when the dressing was changed every 2 days, infusion of imipenem for the antimicrobial prophylaxis of infection, and administration of oxytocin and Chinese herbal medication to promote uterine involution. No systemic infection or dysfunction of internal organs was found during the patient’s stay in the hospital. The patient discharged herself without postpartum complications at 15 days after injury due to financial reasons. At discharge, the superficial second-degree and part of the deep partial thickness burn wounds that were relatively superficial were healed, and less than 10% TBSA deep partial thickness burn wounds remained on the neck, right upper limb, and both legs. No signs of difficulty of healing that could imply the connection of impaired healing and pregnancy were observed. Physical examination results were as follows: blood pressure, 119/76 mmHg; pulse rate, 90 bpm; body temperature, 36.4°C; and respiratory rate, 17 bpm. Laboratory results were as follows: blood routine examination: WBC, 8.76 × 10^9^/L; neutrophil granulocyte, 71.3%; Hgb, 111 g/L; RBC, 4.12 × 10^12^/L; Hct, 0.337; platelet, 359 × 10^9^/L; glutamic pyruvic transaminase, 19 IU/L; glutamic-oxalacetic transaminase, 23 IU/L; alkaline phosphatase, 81 IU/L; TP, 71 g/L; albumin, 36 g/L; urea nitrogen, 3.6 mmol/L; creatinine, 37 μmol/L.

**Table 1:** **Volume of fluid resuscitation (mL)**

**Times**	**Estimated crystalloid and colloid solution**	**Actual infused crystalloid and colloid solution**	**Actual infused crystalloid solution**	**Actual infused colloid solution**	**Urine output**
First 24 h	4,200	2,300	1,500	800	1,985
Second 24 h	2,100	1,100	500	600	3,840
Third 24 h				400	4,245

The solution delivered during surgery (which was excluded from the total fluid resuscitation): 1,000 mL of lactate Ringer’s solution, 500 mL of normal saline, and 600 mL of plasma.

## Discussion

The hemodynamic changes for the severe burn patient in late pregnancy showed specific characteristics. The cardiac output and volume of circulating blood of the pregnant woman started increasing from approximately 6 to 8 weeks and gradually reached a peak at 32~34 weeks of pregnancy, an increase of 30%~45% in total volume (1,500 mL on average). After delivery, the increased cardiac output and volume of circulating blood were gradually restored to the basal level at approximately 2–3 weeks [11]. Although it is well known that intense resuscitation is needed for pregnant women, there are no detailed instructions regarding what is the proper amount of fluid for those patients. There are many resuscitation formulas adopted by different burn units [12], and Ruijin’s formula was used for guiding the burn patients’ resuscitation at our department. Ruijin’s formula is a modification of Evans formula and is more suitable for patients of Asian descent and has been applied in several burn centers of China for more than 50 years. Ruijin’s formula and the Third Military Medical University (TMMU) formula are the most influential formulas in China. Different from the TMMU formula, Ruijin’s formula sets the ratio of crystalloid solution versus colloid solution at 1:1. In this case, the estimated volume of the crystalloid and colloid solution for the first 24 h was 4,200 mL; the actual infusion amount was 4,400 mL (including an intra-operative amount of 2,100 mL). The actual infusion amount of the second 24 h was 1,100 mL, which was 52.38% of the estimated amount (half of 4,200 mL). Considering the extra requirement for the surgery, the actual infusion amount for the first 24 h was much lower than the estimated amount. The urine amount at the first 48 h had reached 50~350 mL/h, which was significantly higher than 0.5~1 mL/kg/h of urine output, which is the general requirement for an effective resuscitation. We believed that the increased cardiac output and body circulating volume, particularly when the baby was delivered, would make the patient more tolerant to fluid loss. Therefore, the infusion volume during shock resuscitation for these maternity patients could be reduced appropriately depending on the urine volume and general condition. Based on our literature search, ours is the first case report to suggest decreasing the infusion volume of fluid resuscitation, particularly after the fetus is delivered. Unfortunately, we only monitored the heart rate, blood pressure, and urine output for the guidance of resuscitation. Indeed, it will be greatly helpful to utilize more hemodynamic parameters to judge the patients’ circulation status and heart function and guide resuscitation.

Pregnancy with burns not only makes treatment for the mother difficult but also threatens the fetus. Within the context of ensuring the survival of the neonate, late pregnancy patients should terminate their pregnancy as early as possible to prevent fetal distress, improve the survival possibility of the fetus, and simplify the management of pregnant women. The correct judgment of pregnant week is crucial. If the gestational week is uncertain and prenatal examination is not performed routinely, the measurement of the physical indexes of the fetus, including the biparietal diameter, head circumference, and height of the femur using B-mode ultrasound, will be useful for the assessment of gestational age and maturity of the fetus. If the fetus is still immature, continuation of pregnancy could be considered under close monitoring, but the fetus is under the risk of drug toxicity due to the treatment strategies. Much literature has indicated that septicemia and sepsis are the main causes of death in pregnant women [8,13], and the administration of antibiotics is inevitable. The US Food and Drug Administration (FDA) has divided antibiotics into five grades (A, B, C, D, and X) according to the possible side effects for the fetus. Penicillins, macrolides, cephalosporins, lincomycin, and clindamycin belong to grade B drugs, which are safe for pregnant women and could be used during pregnancy. Quinolones, itraconazole, and fluconazole belong to grade C drugs, and animal experiments have revealed negative influences on the fetus; therefore, they are selected only when potential benefits exceed potential risks. Silver sulfadiazine could be absorbed from the wounds, and the absorption amount is related to the applied area and duration. Research had reported that the average Ag^+^ concentration in the blood reaches 190 ng/mL at 10 days after application for patients with <35% TBSA, and the Ag^+^ was observed to deposit in the liver and kidneys [14]. Extensive area application of silver sulfadiazine should be avoided for pregnant women with burns because absorption of sulfonamides is related to kernicterus. In our case, silver sulfadiazine was applied after the fetus was delivered. Additionally, imipenem was given after cesarean delivery.

Pregnancy causes tremendous neurological and endocrine changes, including an activated renin-angiotensin-aldosterone system [15] and an increased level of cortisol and the catecholamine adrenaline [16,17]. Heart disease and heart failure following pregnancy-induced hypertension [18,19] and gestational diabetes [20] are not uncommon in pregnant women. It is reasonable to presume that severe burns might aggravate the pathological changes of gravida and result in a deteriorative outcome based on the current knowledge on pathological changes for pregnancy and severe burns. Because related reports and research are scarce, the collection and summarization of more clinical data are needed.

## Conclusions

In summary, the timely termination of pregnancy at the early stage after injury and appropriate modification of the volume of solution for fluid resuscitation based on vital signs and urine output of the patient are two pivotal elements for the success of this case. However, the exact status of hemodynamics, including the heart function and circulation volume, remained uncertain due to the deficiency in invasive monitoring. The collection of more precise data will be very helpful for the decision-making concerning pregnant women with a severe burn injury in the future.

## Consent

Written informed consent was obtained from the patient for publication of this case report and any accompanying images. A copy of the written consent is available for review by the Editor of this journal.
